# Can the Self-Assembling of Dicarboxylate Pt(IV) Prodrugs Influence Their Cell Uptake?

**DOI:** 10.1155/2021/9489926

**Published:** 2021-06-19

**Authors:** Mauro Ravera, Elisabetta Gabano, Elena Perin, Beatrice Rangone, Diego Bonzani, Domenico Osella

**Affiliations:** Dipartimento di Scienze e Innovazione Tecnologica, Università del Piemonte Orientale, Viale Michel 11, Alessandria 15121, Italy

## Abstract

The possibility of spontaneous self-assembly of dicarboxylato Pt(IV) prodrugs and the consequences on their uptake in cancer cells have been evaluated in different aqueous solutions. Four Pt(IV) complexes, namely, (OC-6-33)-diacetatodiamminedichloridoplatinum(IV), **Ace**, (*OC*-6-33)-diamminedibutanoatodichloridoplatinum(IV), **But**, (*OC*-6-33)-diamminedichloridodihexanoatoplatinum(IV), **Hex**, and (*OC*-6-33)-diamminedichloridodioctanoatoplatinum(IV), **Oct**, have been dispersed in i) milliQ water, ii) phosphate buffered saline, and iii) complete cell culture media (RPMI 1640 or DMEM) containing fetal bovine serum (FBS). The samples have been analyzed by dynamic light scattering (DLS) to measure the size and distribution of the nanoparticles possibly present. The zeta potential offered an indication of the stability of the resulting aggregates. In the case of the most lipophilic compounds of the series, namely, **Oct** and to a lesser extent **Hex**, the formation of nanosized aggregates has been observed, in particular at the highest concentration tested (10 *μ*M). The cell culture media had the effect to disaggregate these nanoparticles, mainly by virtue of their albumin content, able to interact with the organic chains via noncovalent (hydrophobic) interactions. For **Oct**, at the highest concentration employed for the uptake tests (10 *μ*M), the combination between passive diffusion and endocytosis of the self-assembled nanoparticles makes the cellular uptake higher than in the presence of passive diffusion only. During the study of cellular uptake on A2780 ovarian cancer cells pretreated with cytochalasin D, a statistically significant inhibition of endocytosis was observed for **Oct**. In these experimental conditions, the relationship between uptake and lipophilicity becomes almost linear instead of exponential. Since **Oct** anticancer prodrug is active at nanomolar concentrations, where the aggregation in culture media is almost abolished, this phenomenon should not significantly impact its antiproliferative activity.

## 1. Introduction

**Figure 1:**
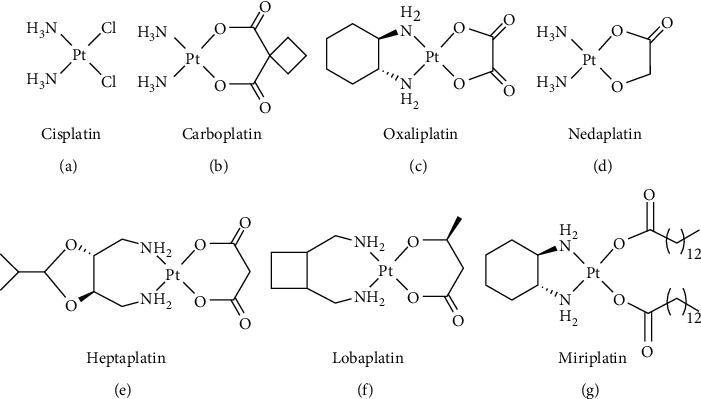
Chemical structures of the clinically approved and marketed Pt(II) anticancer drugs.

Targeted- and immuno-therapy represent a new roadmap in cancer treatment to obtain high therapeutic effects with low systemic toxicity [1]. Nevertheless, the traditional cytotoxic drugs (especially the DNA-damaging ones) maintain an immeasurable clinical value against several aggressive solid tumors. Among these cytotoxic drugs, three Pt(II) complexes have been worldwide approved, namely, cisplatin, carboplatin, and oxaliplatin. In addition, nedaplatin, heptaplatin, and lobaplatin have been regionally approved in Japan, China, and South Korea, respectively [2]. Finally, the very lipophilic platinum complex miriplatin was approved in Japan for lipiodolization in the treatment of hepatocellular carcinoma [3]. The structures of these Pt(II) drugs are shown in Figure 1. Interestingly enough, cisplatin is still the main component in several clinical trials (note: the NIH-registered clinical trials involving cisplatin in various parts of the world can be obtained by using the search tools from http://www.clinicaltrials.gov/). Unfortunately, Pt(II)-based drugs have numerous drawbacks including low bioavailability and stability, severe side effects, and inherent or acquired resistance [4].

**Figure 2:**
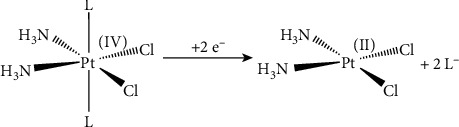
Scheme of the 2e^−^ reduction of a cisplatin-based Pt(IV) complex.

In an attempt to overcome these shortcomings, Pt(IV) complexes have been proposed as an alternative to Pt(II) drugs. They exhibit higher kinetic inertness, lowering the incidence of unwanted off-reactions, thus ameliorating some side effects and rendering them suitable for oral administration. Pt(IV) complexes act as prodrugs of the corresponding Pt(II) congeners, being activated in cells by reductive elimination of the axial ligands (favored by the hypoxic and then reducing tumor microenvironment) to release the active Pt(II) complex (Figure 2) [5, 6].

**Figure 3:**
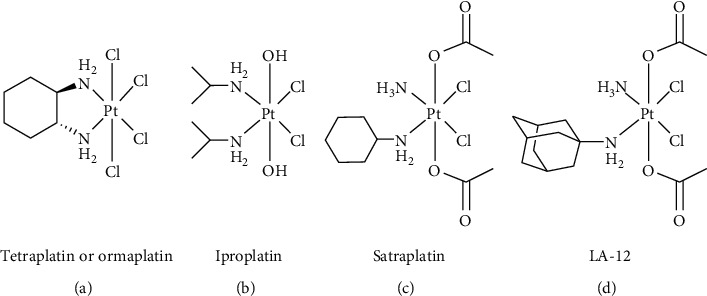
Chemical structures of the Pt(IV) complexes cited in the text. (a) Tetraplatin or ormaplatin. (b) Iproplatin. (c) Satraplatin. (d) LA-12

Since the Pt(IV) prodrugs are obtained from the oxidation of the corresponding Pt(II) congeners (usually with chlorine or hydrogen peroxide), the first two complexes that underwent clinical trials were tetraplatin (or ormaplatin), bearing two chlorido ligands in the axial position, and iproplatin, bearing two hydroxido ligands in axial position (Figure 3). Tetraplatin exhibited too high systemic toxicity, whereas iproplatin was poorly active. The ease of reduction of Pt(IV) complexes depends mainly on the nature of the axial ligands and occurs in the order Cl^−^ > acetato > OH^−^. Hence, dichlorido complexes are reduced too fast and dihydroxido complexes are reduced too slowly to exert an optimal pharmacological activity [7]. The carboxylation process of dihydroxido Pt(IV) synthons afforded a plethora of dicarboxylato complexes having reduction potential in the proper biological window [8]. Among them, the diacetato prodrugs satraplatin (or JM216) and LA-12 underwent several clinical trials, unfortunately without having obtained full approval so far. An inspection on their structures (Figure 3) shows that the high lipophilicity of such diacetato Pt(IV) prodrugs, allowing an efficient cellular uptake, has been always obtained by using bulky amines as carrier ligands (cyclohexylamine and adamantylamine, respectively).

**Figure 4:**
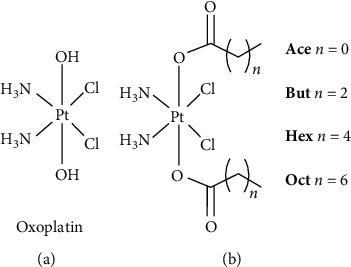
(a) Chemical structure of the precursor oxoplatin and (b) that of the compounds under investigation (**Ace**, But, **Hex**, and **Oct**).

Alternatively, fatty acids (FAs) could be employed with the same aim of increasing lipophilicity, thus maintaining the basal cisplatin skeleton (i.e., starting from oxoplatin, Figure 4, for the carboxylation reaction). This offers some advantages being the starting material (cisplatin) readily available and its mechanism of action (once released upon Pt(IV)–Pt(II) reduction) relatively well understood. For a very homogeneous series of FAs Pt(IV) complexes, their potency (1/IC_50_) can be quite satisfactory related to an electronic factor (i.e., their reduction potential or charge at the Pt atom) and to a lipophilicity factor (i.e., the partition coefficient between octanol and water log *P*_o/w_, or, more simply, the MW or the length of the carboxylic chains) [9, 10]. The latter parameters have little effect on the electronic density of the Pt(IV) core. On the contrary, since for such Pt(IV) conjugates the main (if not the only) cellular uptake mechanism is the passive diffusion, their cellular uptake results to be proportional to the length of the carboxylic chains, which governs the log *P*_o/w_ [11]. In the hopeful hypothesis to use these dicarboxylato Pt(IV) complexes as single-molecule antitumor prodrugs for oral assumption (*per os*), the length of each carbon chain should be limited (C_2_–C_8_); otherwise, their water solubility falls and their gastrointestinal absorption becomes poorly efficient, thus lowering their peak concentration in the circulating blood [12, 13]. On the other hand, a myriad of Pt(IV) complexes bearing carboxylate ligands have been synthesized and tested, including high molecular weight [14] or polyunsaturated FAs [15], and several organic drugs (bearing a reactive carboxylic group for conjugation, such as the COX inhibitors aspirin and flurbiprofen [16–18]). All these extremely lipophilic Pt(IV) conjugates spontaneously self-assemble in aqueous solution and this improves the *in vitro* cellular uptake, adding to the passive diffusion the very effective endocytosis process undergone by the corresponding nanoaggregates. These nanoparticles are generally cloaked or inserted in PEGylated materials in order to increase their stability, solubility, and controllable release profile [19–22].

On this background, we investigated whether the simple dicarboxylato cisplatin-based **Ace**, **But**, **Hex**, and **Oct** (Figure 4), originally tested as single-molecule antiproliferative prodrugs [12], are able to self-assemble in aqueous solution to generate carrier-free nanostructure and whether this possible phenomenon could have any significance in the experimental conditions employed in the preclinical tests.

## 2. Materials and Methods

The Pt(IV) complexes (*OC*-6-33)-diacetatodiamminedichloridoplatinum(IV), **Ace**, (*OC*-6-33)-diamminedibutanoatodichloridoplatinum(IV), **But**, (*OC*-6-33)-diamminedichloridodihexanoatoplatinum(IV), (**Hex**, and (*OC*-6-33)-diamminedichloridodioctanoatoplatinum(IV), **Oct**, were prepared from oxoplatin according to published procedures [12, 23, 24]. Briefly, a suspension of oxoplatin in DMF was reacted with an excess of the appropriate anhydride (for **Ace**, **But**, and **Hex**) or acyl chloride (for **Oct**) to obtain a solution of the *bis*(carboxylato)-Pt(IV) complex. The volume of the solution was reduced, and diethyl ether was added to precipitate the product. Characterization and determination of the purity of the complexes was performed by means of the usual analytical techniques (i.e., elemental analysis, HPLC, ESI-MS, and multinuclear NMR).

Dynamic light scattering (DLS) and zeta potential analyses were performed in 10 mM KNO_3_ (after pre-treatment with HCl 0.5 M and following centrifugation) at 37°C with a Malvern Zetasizer Nano ZS (Malvern Instruments Ltd., Malvern, UK) at a fixed scattering angle of 173°, using a He–Ne laser and DLS software for Windows (version 6.11, Malvern, UK). The preparation of the solutions and corresponding DLS measurements were performed at least in triplicate by two independent operators to mediate the effect of the manipulation procedures on the results. Each value reported in the figures is the average of at least six measurements.

### 2.1. Preparation of the Solutions

Mother solutions of the four complexes were prepared in DMSO and diluted to 1 mM. Aliquots of these solutions were diluted in one of the following aqueous media: i) milliQ water (H_2_O), ii) phosphate buffered saline (PBS), iii) RPMI 1640 cell culture medium, or iv) Dulbecco's Modified Eagle Medium (DMEM); the cell culture medium was supplemented with 10% v/v fetal bovine serum (FBS). For each solution, five samples were prepared with concentrations of 0.1, 0.5, 1, 5, and 10 *μ*M and a fixed final 1.0% v/v organic cosolvent amount by adding suitable volumes of pure DMSO. Finally, three PBS solutions containing 1.0% v/v DMSO were prepared with **Oct** (0.1 *μ*M) alone, bovine serum albumin (BSA, 35 *μ*M) alone, and a mixture of both compounds.

### 2.2. Cellular Uptake

A2780 cells (ICLC HTL98008, Interlab Cell Line Collection, Genova, Italy) were seeded in T25 flasks and allowed to grow until around 80% confluence. Then, the treatment was performed for 2 h with the complexes under investigation (10 *μ*M) in complete RPMI 1640 medium (with 10% FBS). At the end of the exposure, cells were washed three times with PBS, detached from the Petri dishes using 0.05% Trypsin 1X + 2% EDTA (HyClone, Thermo Fisher) and harvested in fresh complete medium. An automatic cell counting device (Countess®, Life Technologies) was used to measure the number of cells and their mean diameter for every count. In the experiments with the inhibitor cytochalasin D, pre-incubation for 30 min in RPMI 1640 with 10 *μ*M cytochalasin D was followed by a 10 *μ*M treatment with the Pt(IV) compounds for 2 h in RPMI 1640. Treated cells were transferred into a borosilicate glass tube and centrifuged at 1100 rpm for 5 min at room temperature. The supernatant was carefully removed by aspiration, while about 200 *μ*L of the supernatant was left to limit the cellular loss. Cellular pellets were stored at −20°C until mineralization and determination of the Pt content. Platinum content determination was performed by inductively coupled plasma-mass spectrometry (ICP-MS, Thermo Optek X Series 2). Mineralization of the samples was performed by the addition of 70% w/w HNO_3_ to each sample, followed by incubation for 1 h at 60°C in an ultrasonic bath. Before the ICP-OES measurements, the HNO_3_ was diluted to a final 1.0% v/v concentration. A Pt standard stock solution of 1000 mg·L^−1^ was diluted in 1.0% v/v nitric acid to prepare calibration standards. Instrumental ICP-MS settings were optimized in order to yield maximum sensitivity for platinum. For quantitative determination, the most abundant isotopes of platinum and indium (used as internal standard) were measured at *m/z* 195 and 115, respectively. The level of Pt found in cells after drug treatment and normalized upon the cell number (cellular Pt uptake) was expressed as ng Pt per 10^6^ cells.

## 3. Results and Discussion

**Figure 5:**
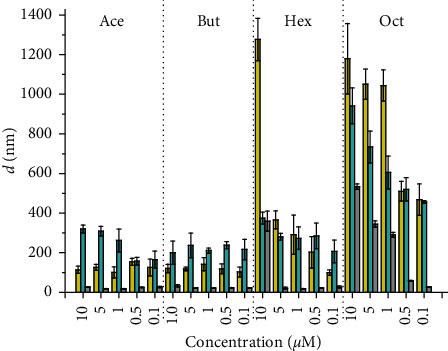
Hydrodynamic radii, *d* (nm), measured by dynamic light scattering (DLS) on the mixtures under investigation containing **Ace**, **But**, **Hex**, and **Oct** at 10, 5, 1, 0.5, and 0.1 *μ*M concentrations in 1.0% DMSO/aqueous solutions. Color code: yellow = milliQ water; cyan = phosphate buffered saline, PBS; grey = RPMI 1640. Data are means ± standard deviation (sd) of at least six measurements (statistical analysis was omitted for clarity, see Supplementary Materials).

Dispersions of the title Pt(IV) complexes were prepared in four different media: i) milliQ water (H_2_O), ii) phosphate buffered saline (PBS), iii) RPMI 1640, and iv) DMEM cell culture media, the last two supplemented with 10% fetal bovine serum (FBS) as in the culture media used in the *in vitro* preclinical experiments [25]. The samples have been prepared from concentrated mother solutions in DMSO, diluted in the aqueous media to five final concentrations (0.1, 0.5, 1, 5, and 10 *μ*M). The % of DMSO was kept in every solution at 1.0% v/v. The mixtures were analyzed by dynamic light scattering (DLS) to obtain the size (hydrodynamic radii, d) and the distribution of the nanoparticles present (polydispersity index, PDI).  Figure 5 shows a condensed representation of the results (see also Figures S2–S5, Supplementary Materials).

In H_2_O, both **Ace** and **But** show the presence of nanoparticulate with *d* 100 nm, almost independently of the concentration. On the contrary, **Hex** and **Oct** showed rather higher values of *d*, with a trend that increases as the concentration of the complexes increases.

The presence of salts (PBS) conveyed the mean *d* values between 200 and 400 nm for **Ace**, **But** and **Hex**, buffering the differences with concentration. Only in the case of **Oct** is the correlation between *d* and concentration maintained.

In the presence of the complete cell culture medium RPMI 1640 (but also DMEM, see Figure S5, Supplementary Materials), a generalized, dramatic decrease of *d* was observed. For **Ace** and **But** at all concentrations, as well as for **Hex** at concentration ≤5 *μ*M, *d* was always indistinguishable from the background (RPMI 1640 and DMEM alone contain particles having *d* between 20 and 40 nm). On the contrary, **Oct** maintained values of *d* between 300 and 500 nm at the highest concentrations (1–10 *μ*M), whereas the disaggregation of nanoparticles was almost complete at the lowest ones where *d* values dropped to the background ones.

The average uniformity of the particle solution was evaluated with the PDI: samples with PDI values ≤ 0.1 are considered highly monodisperse, whereas values of 0.1–0.4 and > 0.4 (up to 1) are indicative of moderately and highly polydisperse samples, respectively [26]. The PDI values obtained in the present work ranged from 0.8 to 1.0 in H_2_O, from 0.6 to 1.0 in PBS, and from 0.3 to 0.5 in both culture media without any trend.

The different mixtures have a zeta potential ranging between +1 and −16 mV, as expected for aggregates derived from neutral molecules. As a rule of thumb, nanoparticles with a zeta potential between −30 mV and +30 mV are considered electrostatically unstable and disposed to aggregation due to the dominant attractive van der Waals interactions [27].

The striking effect of complete cellular culture media on the aggregation of the title compounds (and hence the corresponding DLS diameter of the generated nanoparticles) can be interpreted on the basis of the seminal work of Johnstone and Lippard, showing as cisplatin-based asymmetric FA-succinato-Pt(IV) conjugates interact with human serum albumin (HSA). Such noncovalent interaction increases in strength as the aliphatic tail increases: among the complexes analyzed, it is very feeble for C_2_, and rather intense for C_16_ (the highest chain considered). In the latter case, the HSA-Pt(IV) adduct, having a 1 : 1 stoichiometry, results in being so robust to be isolated by fast liquid chromatography [14]. There is a large analogy between HSA and bovine serum albumin (BSA), the latter being the main component of FBS, which in turn represents 10% v/v in both the complete media employed (RPMI 1640 and DMEM). Thus, the interaction between Pt(IV) conjugates and BSA should be able to disassemble the nanoparticles generated by aggregation.

**Figure 6:**
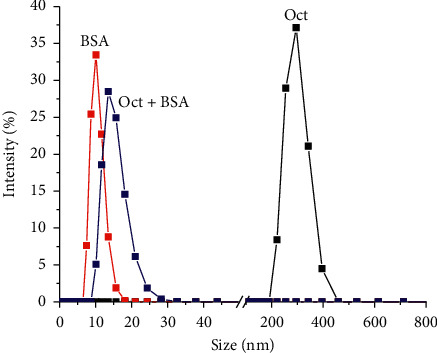
Size distribution curves obtained from DLS data (PBS, 0.1% DMSO): (black line) **Oct** (0.1 *μ*M), (red line) BSA (35 *μ*M), and (blue line) **Oct** (0.1 *μ*M) + BSA (35 *μ*M).

In order to unambiguously demonstrate the role of BSA in disaggregating the nanoparticles, DLS diagrams were recorded for PBS solutions containing **Oct** (0.1 *μ*M) alone, BSA alone (35 *μ*M) [25], and a mixture of both. The DLS peak around 400 nm observed for **Oct** alone shifted around 15 nm in the presence of BSA, a value for the possible adduct BSA-**Oct** very closed at that recorded for BSA alone (ca. 10 nm), values in tune with the range of literature data for monomeric BSA [28] (Figure 6).

**Figure 7:**
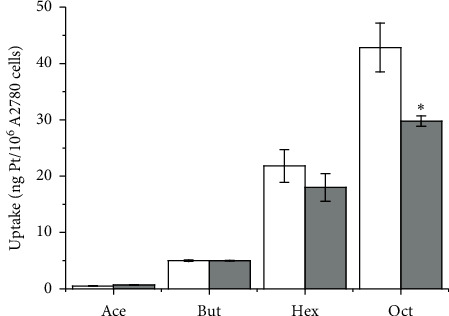
Pt uptake on A2780 ovarian cancer cells treated for 2 h with the title compounds (10 *μ*M) without (white columns) or with (grey columns) 30 min pre-treatment with cytochalasin *D*. Data are means ± sd of at least six independent replicates and were compared by means of a one-way ANOVA-Tukey test (no indication = not significant; ^*∗*^*p* < 0.05).

In order to understand whether endocytosis of self-assembled nanoparticles plays a role at the high concentrations (10 *μ*M), which are generally employed for the Pt uptake experiments (evaluated by ICP-MS) on cancer cells A2780 ovarian cancer cells were challenged with each title Pt(IV) complex in the absence and in the presence of cytochalasin D. The resulting Pt cellular uptake is reported in Figure 7.

As a nanoparticle meets a cell, it is generally internalized by means of endocytosis. Endocytosis can be divided into pinocytosis, which involves the uptake of fluids and molecules within small vesicles, and phagocytosis, which is responsible for engulfing large particles. Pinocytosis can be dependent on the clathrin coat (clathrin-mediated endocytosis) or not (clathrin-independent endocytosis) [29]. There is a variety of approaches to inhibit different forms of endocytosis in order to define the exact uptake mechanism. This is not the aim of the present paper, and a simpler approach by using cytochalasin D has been employed. This cell permeable fungal toxin can depolymerize actin filaments and can therefore be used to study actin-dependent uptake mechanisms. However, this toxin affects almost all endocytic pathways, because inhibitors of actin polymerization have indirect effects on any forms of endocytosis [30].

As a consequence of cytochalasin D pre-treatment, a limited or statistically significant inhibition of cellular uptake of **Hex** and **Oct**, respectively, was observed. On the contrary, the behavior of **Ace** and **But** was not significantly affected by such a pre-treatment.

**Figure 8:**
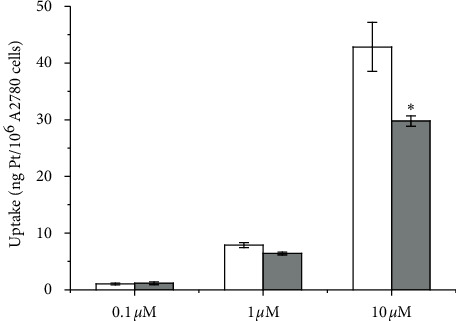
Uptake on A2780 ovarian cancer cells treated for 2 h with **Oct** at three different concentrations, without (white columns) or with (grey columns) pre-treatment with cytochalasin *D*. Data are means ± sd of at least six independent replicates and were compared by means of a one-way ANOVA-Tukey test (no indication = not significant; ^*∗*^*p* < 0.05).

Importantly, this effect is clearly visible at [**Oct**] = 10 *μ*M, whereas, at lower concentrations, cytochalasin D has negligible or no effect on the cell uptake of the most lipophilic complex of the series (Figure 8).

Oldfield et al. found a nonlinear correlation between log *P*_o/w_ and Pt cellular uptake of several Pt(IV) complexes. This correlation was defined as a “nonlinear upward trend.” Both parabolic and exponential relationships resulted statistically valid, but the authors considered more realistic the latter [31, 32]. The four Pt(IV) complexes studied here exhibit a wide range of lipophilicity: their log *P*_o/w_ spans about 6 log units (log *P*_o/w_**Ace** = −1.92, **But** = −0.39, **Hex** = 1.14, and **Oct** = 4.1) [11, 12].

**Figure 9:**
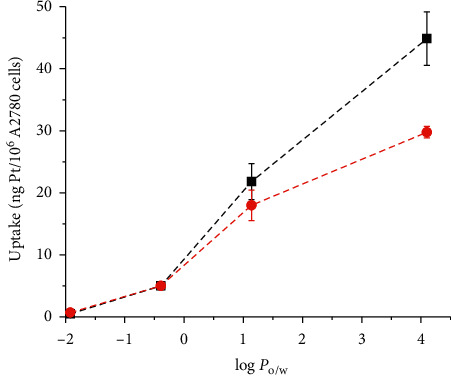
Relationship between log *P*_o/w_ and the uptake on A2780 ovarian cancer cells treated for 2 h with 10 *μ*M concentrations of **Ace**, **But**, **Hex**, and **Oct**, measured without (black line and symbols) or with (red line and symbols) pre-treatment with cytochalasin D.

A similar behavior of uptake as a function of log *P*_o/w_ was observed here (Figures 7 and 9 ). Interestingly enough, using the data of uptake obtained after the inhibition of endocytosis by cytochalasin D pre-treatment, the correlation becomes nearer to linearity (Figure 9). This indicates (at least for these Pt(IV) conjugates) that the additional effect of the endocytosis of self-assembled aggregates at the high concentration (10 *μ*M) necessarily employed for any uptake study seems to be involved in the “nonlinear upward trend” of uptake as a function of log *P*_o/w_.

## 4. Conclusions

Summing up the results obtained in this study, some conclusions can be drawn from the reported experiments:

1. In the case of the most lipophilic compounds of the series, the formation of nanosized aggregates has been observed, in particular at the highest concentration tested. However, they are characterized by low zeta potentials, causing low stability and high dispersity. Most importantly, the complete cell culture media (by virtue of the presence of BSA) have the effect of disaggregating the nanoparticles.
2. At high concentrations, the combination between passive diffusion (at the molecular level) and (more efficient) endocytosis of the aggregates makes the Pt uptake higher than that expected for passive diffusion only. However, for compounds active at nanomolar concentrations, as **Oct** [12, 33], this effect should not affect the observed antiproliferative activity. Indeed, the 100 nM concentration of **Oct** employed for DLS investigation represents 50 × IC_50_ on the cisplatin sensitive A2780 tumor cell line.
3. Studying lipophilic Pt(IV) complexes, caution must be paid to the interpretation of the results of biological tests, since, according to the experimental conditions, they can turn from single molecules to self-aggregates.

## Data Availability

The data used to support the findings of this study are included within the article and the Supplementary Materials file.

## Supplementary Materials

Supplementary Materials Detailed hydrodynamic radii measured by dynamic light scattering (DLS) on the mixtures under investigation containing **Ace**, **But**, **Hex**, and **Oct** in water, PBS, RPMI 1640, and DMEM solutions.
