# Supplementary material for: Can the Self-Assembling of Dicarboxylate Pt(IV) Prodrugs Influence Their Cell Uptake?
Source: Bioinorg Chem Appl. 2021 Jun 19;2021:9489926. doi: 10.1155/2021/9489926 (PMC8235969; doi:10.1155/2021/9489926)
Supplement: Supplementary Materials — Detailed hydrodynamic radii measured by dynamic light scattering (DLS) on the mixtures under investigation containing Ace, But, Hex, and Oct in water, PBS, RPMI 1640, and DMEM solutions. [file 9489926.f1.pdf]

May the self-assembling of dicarboxylate Pt(IV) prodrugs influence their cell uptake?

Mauro Ravera, Elisabetta Gabano, Elena Perin, Beatrice Rangone, Diego Bonzani, and Domenico Osella

Dipartimento di Scienze e Innovazione Tecnologica, Università del Piemonte Orientale, Viale Michel 11, 15121 Alessandria (Italy)

Correspondence should be addressed to Domenico Osella; domenico.osella@uniupo.it

SUPPLEMENTARY MATERIALS

Figure S1 Chemical structure of the compounds under investigation.

Figure S2 Hydrodynamic radii, d (nm), measured by dynamic light scattering (DLS) on the mixtures under investigation containing **Ace**, **But**, **Hex** and **Oct** 1% DMSO / water solutions.

Figure S3 Hydrodynamic radii, d (nm), measured by dynamic light scattering (DLS) on the mixtures under investigation containing **Ace**, **But**, **Hex** and **Oct** 1% DMSO / PBS solutions.

Figure S4 Hydrodynamic radii, d (nm), measured by dynamic light scattering (DLS) on the mixtures under investigation containing **Ace**, **But**, **Hex** and **Oct** 1% DMSO / RPMI 1640 solutions.

Figure S5 Hydrodynamic radii, d (nm), measured by dynamic light scattering (DLS) on the mixtures under investigation containing **Ace**, **But**, **Hex** and **Oct** 1% DMSO / DMEM solutions.

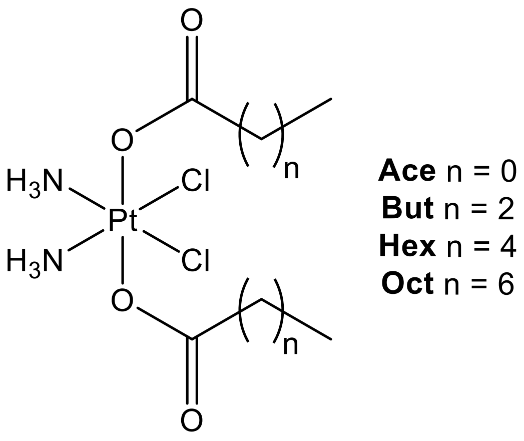

Figure S1. Chemical structure of the compounds under investigation.

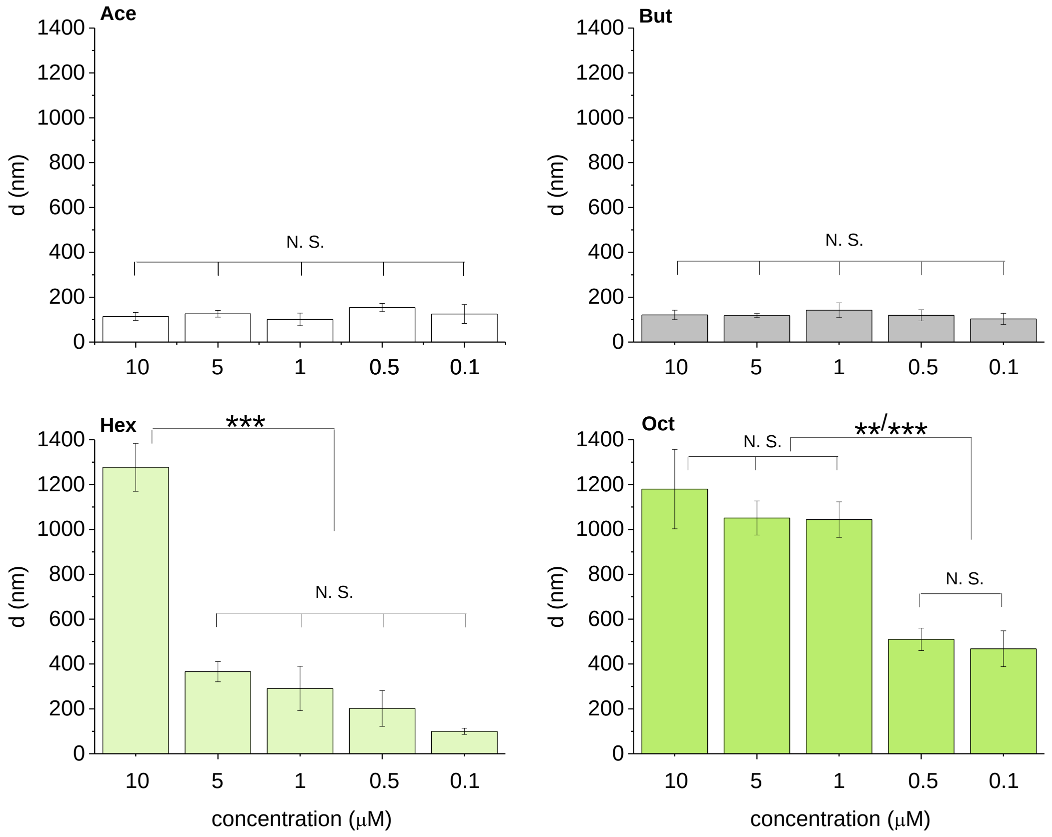

Figure S2. Hydrodynamic radii, d (nm), measured by dynamic light scattering (DLS) on the mixtures under investigation containing **Ace**, **But**, **Hex** and **Oct** at 10, 5, 1, 0.5, and 0.1 μM concentrations in 1% DMSO / water solutions. Data are means ± standard deviations (sd) of at least six measurements and compared by means of a one-way ANOVA-Tukey test (*** $p < 0.001$, ** $p < 0.01$, ** $p < 0.05$, N.S. = not significant).

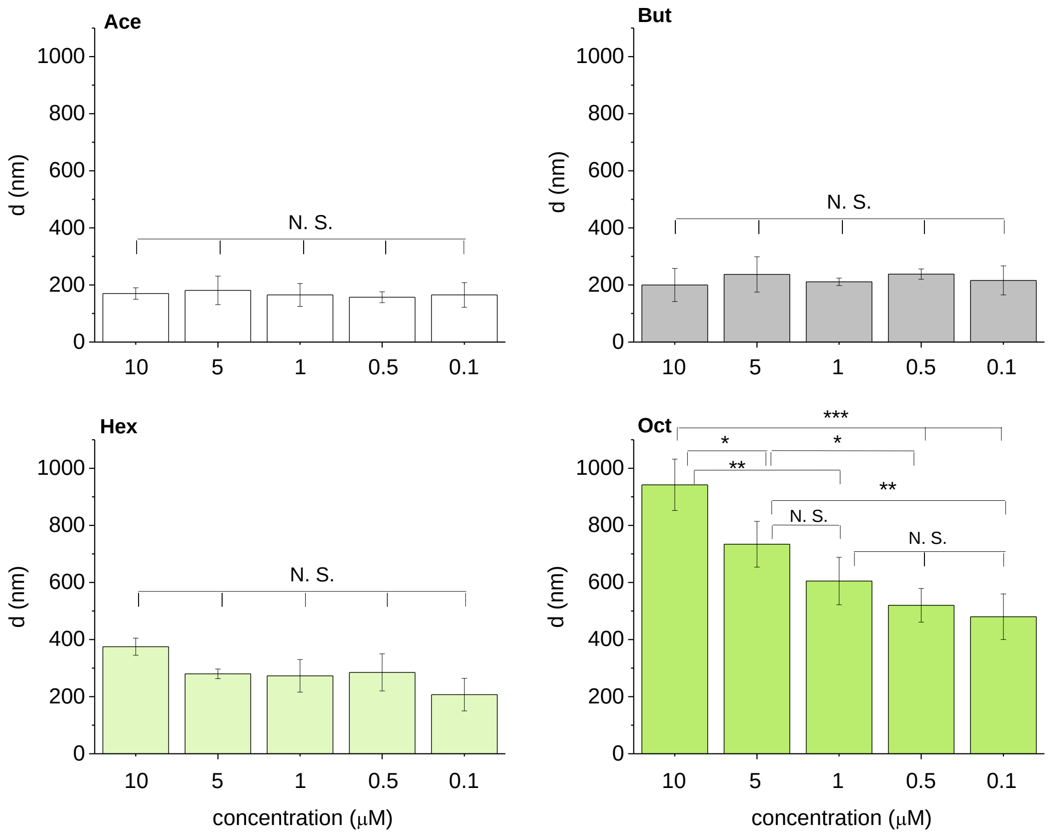

Figure S3. Hydrodynamic radii, d (nm), measured by dynamic light scattering (DLS) on the mixtures under investigation containing **Ace**, **But**, **Hex** and **Oct** at 10, 5, 1, 0.5, and 0.1 μM concentrations in 1% DMSO / PBS solutions. Data are means ± sd of at least six measurements and compared by means of a one-way ANOVA-Tukey test (*** $p < 0.001$, ** $p < 0.01$, ** $p < 0.05$, N.S. = not significant).

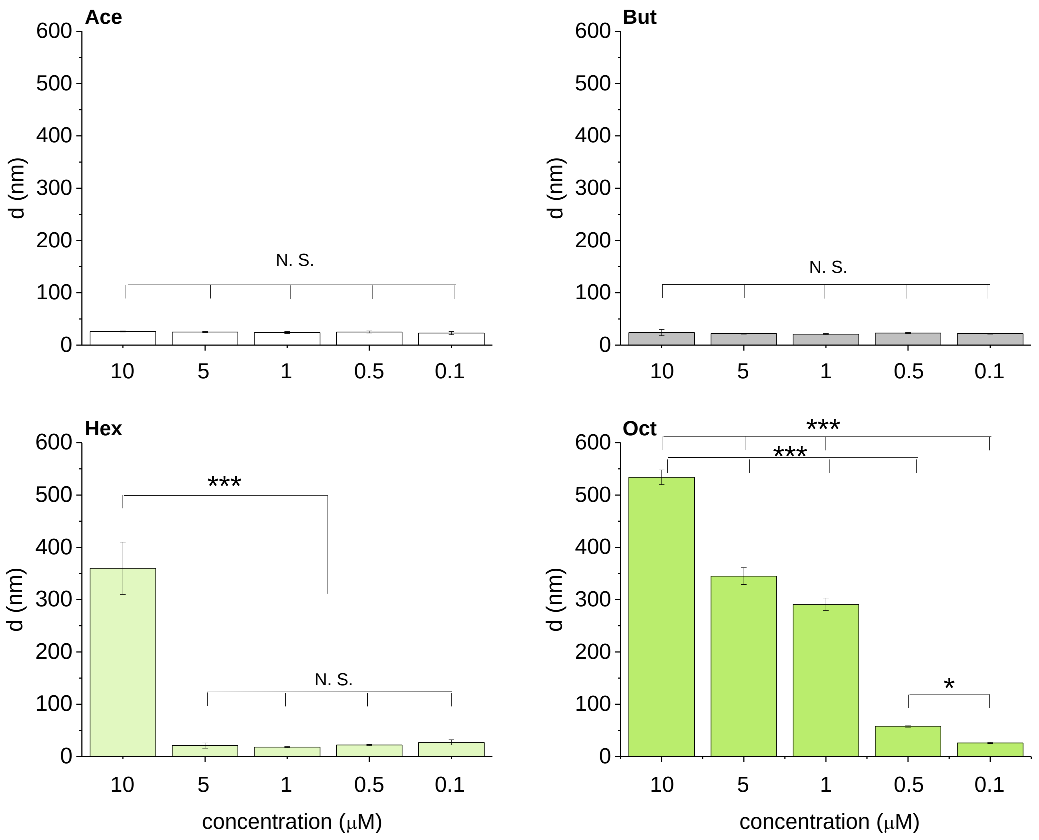

Figure S4. Hydrodynamic radii, d (nm), measured by dynamic light scattering (DLS) on the mixtures under investigation containing **Ace**, **But**, **Hex** and **Oct** at 10, 5, 1, 0.5, and 0.1 μM concentrations in 1% DMSO / RPMI solutions. Data are means ± sd of at least six measurements and compared by means of a one-way ANOVA-Tukey test (*** $p < 0.001$, ** $p < 0.01$, ** $p < 0.05$, N.S. = not significant).

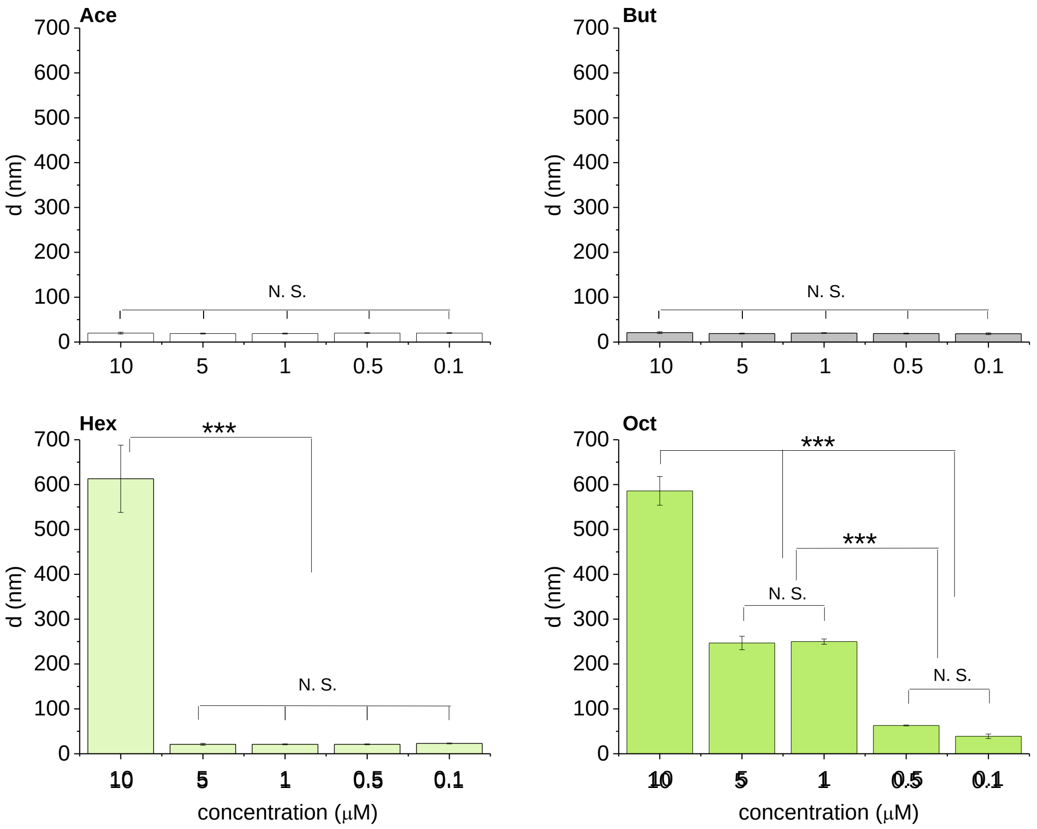

Figure S5. Hydrodynamic radii, d (nm), measured by dynamic light scattering (DLS) on the mixtures under investigation containing **Ace**, **But**, **Hex** and **Oct** at 10, 5, 1, 0.5, and 0.1 μM concentrations in 1% DMSO / DMEM solutions. Data are means ± sd of at least six measurements and compared by means of a one-way ANOVA-Tukey test (*** $p < 0.001$, ** $p < 0.01$, ** $p < 0.05$, N.S. = not significant).
